# A partial validation of the WHOQOL-OLD in a sample of older people in South Africa

**DOI:** 10.3402/gha.v8.28209

**Published:** 2015-10-06

**Authors:** Lizanle Van Biljon, Petrus Nel, Vera Roos

**Affiliations:** 1 Africa Unit for Transdisciplinary Health Research, Faculty of Health Sciences, North-West University, Potchefstroom, South Africa; 2 Industrial Psychology, Faculty of Economic and Management Sciences, University of the Free State, Bloemfontein, South Africa

**Keywords:** quality of life, older people, long-term care facility, psychometric properties, WHOQOL-OLD measuring instrument

## Abstract

**Background:**

This paper describes the psychometric properties of the WHOQOL-OLD, an add-on module to the World Health Organization's Quality of Life measure for older people in a South African sample. The WHOQOL-OLD module was further condensed into three short versions which contain the best items of the original module. The psychometric properties associated with the three short versions of the WHOQOL-OLD are also described.

**Method:**

Data were collected from Afrikaans-speaking older people (*n*=176) residing in long-term care facilities in Potchefstroom, situated in the North-West province of South Africa. The mean age of participants was 77 years (SD=8.1). Fifty participants were males and 126 were females. All reported average-to-good health and cognitive ability.

**Results:**

The current study found encouraging results related to the original factor structure of the WHOQOL-OLD as well as the three short versions of this instrument. Results stemming from the data of the current sample seem to be a good fit with the original factor structure of the WHOQOL-OLD. The reliabilities associated with the various sub-dimensions point to a reliable instrument.

**Conclusions:**

The WHOQOL-OLD with its 24 items or any of the three short versions of this instrument can, therefore, be utilised in a South African context (Version 3 of the short versions seems to be the better fitting version).

The transition to an older age structure (also known as population ageing) is a consequence of gains in average life expectancy and long-term downward trends in fertility (1). The work of Joubert and Bradshaw (2) has shown that population ageing was formerly experienced as a gradual process by more developed countries. Currently developing countries conform to similar demographic trends and are ageing faster than developed countries. As a developing country, South Africa is one of the most rapidly ageing populations in Africa Westaway et al. (3). Eighty-six percent of all older persons in the southern part of Africa reside in South Africa, according to the Population Reference Bureau (4).

With the swift increase in the number of older persons, an increasing demand for long-term care facilities has arisen. According to South African legislation, a long-term care facility can be described as ‘a building or other structure used primarily for the purposes of providing accommodation and of providing 24-hour service to older persons’ (5). Pre-1994, socio-political influences have exerted a major impact on the availability of long-term care for all older people in South Africa. The majority of facilities were only made available for white older people under apartheid rule (6). An audit by the Department of Social Development found that 79% of these facilities are concentrated in metropolitan areas or small urban areas (also known as formal areas). A mere 5% of long-term care facilities are situated in squatter areas or informal areas. There seems to be more of a distribution in rural areas (16%). The majority of these facilities are currently still occupied predominantly by white older people (6).

Long-term care facilities are a well-liked preference for older people as it provides them with safety and security. Concepts such as service, care, comfort, and socialisation form part of what these facilities have to offer (7). However, the conversion from living independently to living in an institution is challenging for some older individuals. Various intra- and interpersonal forfeits are required when adapting in a new living environment (8). Physical living space is often reduced and privacy is often compromised. Individual circumstances determine the fostering- or frustrating effect of institutionalised living. The latter has a great impact on the quality of life (QoL) of older people. In long-term care facilities, QoL is furthermore found to be a significant predictor of mortality and physical dependence (9).

The properties of QoL have been studied widely across many disciplines with limited indication of its exact meaning (10). For some time now, there seems to have been a growing recognition that the QoL of older people is complex and the study thereof requires greater transparency in terms of context, population dynamics and research aims, methodology and theoretical grounding (11). The definition of QoL also brings to the fore certain controversies as there is little consensus on how QoL ought to be defined (12, 13). The definition proposed by the World Health Organization Quality of Life group (14) is adoptedfor the purpose of this study. The group holds that QoL is

a multi-dimensional concept which involves an individual's perception of their position in life in the context of the culture and value systems in which they live and in relation to their goals, expectations, standards and concerns.

QoL research in South Africa has largely demonstrated a focus on specific societies (15–17); socio-economic and healthcare perspectives (15, 16, 18). There seems to be evidence of various qualitative studies amongst older people living in long-term care facilities (19–23). However, there is a lack of QoL measurements developed for and within the elderly population (24). Internationally various measures of QoL in older age groups have become increasingly popular (25, 26), including Older People's Quality of Life Questionnaire (OPQOL), the 19-item Control, Autonomy, Satisfaction and Pleasure-Questionnaire (CASP-19) and the older people version of the World Health Organization's Quality of Life Questionnaire – version for older people (WHOQOL-OLD). This study took particular interest in the WHOQOL-OLD instrument.

The work of Fang et al. (27) further condensed the WHOQOL-OLD module by establishing three short-form versions of the module. The short forms contain the best items of the original module, but they are much shorter and demonstrate good internal consistency and criterion validity. Older adults with poor vision or physical disabilities or serious illness may find it less problematic to read and complete the shorter version of the questionnaire (27). These researchers suggested that

more studies on the validation of these three versions of the short-form WHOQOL-OLD module will be necessary with new data sets in order to allow implementation in future international studies. (27, p. 77)

On par with studies in other countries such as Norway (24), Brazil (28), and the United Kingdom (27), the aim of this study is to describe the reliability of the WHOQOL-OLD and the short versions of the module by means of analysing the metric properties thereof based on research conducted in a South African sample of older people (aged 60 years and older) residing in long-term care facilities.

## Problem statement

An audit of long-term care facilities by the Department of Social Development (6) stipulated that the QoL of older people in these settings is ill-defined and undetermined. A failure to operationalise the concept of QoL adequately for older people will endanger welfare proposals as well as comparisons with other populations. The use of lengthy questionnaires has been found to be ineffective and futile amongst older people as they are opposed to the completion of comprehensive forms to report on their health or psychological status, as this is often tiring and inconvenient to them (27). The WHOQOL-OLD is a 24-item, 6-facet instrument with cross-cultural reliability (29, 30). The instrument has won ground internationally as a concise add-on instrument which yields valuable information pertaining to older people's QoL when used in conjunction with the generic WHOQOL instruments (which has not been validated explicitly for use on older people). Three short versions of the original 24-item instrument, consisting of six items each, have been proposed (27). Reliable use of the WHOQOL-OLD or its short forms amongst older people within a South African sample will yield a concise measure of older people's rating of their QoL in long-term institutions and furthermore provide caregivers, management, and even policy makers with a more holistic idea of what older people need in order to have QoL. The research question is, therefore, whether or not the WHOQOL-OLD and the shorter versions thereof can be used as a reliable instrument for measuring QoL of older people living in long-term care facilities in South Africa.

## Aims and objectives

The aim is a partial validation (i.e. the assessment of the factor structure and the internal consistency reliability) of the WHOQOL-OLD and its shorter versions. To achieve this aim, two objectives will guide the current study.

The first objective of the current study is to determine the psychometric properties of the WHOQOL-OLD amongst older people in long-term care facilities in Potchefstroom, South Africa. Second, the current study aims to determine the metric properties associated with three short versions of the WHOQOL-OLD with a view to determining which short version would be most suitable when used in conjunction with the WHOQOL-100 or Bref (the latter is a more elaborate QoL instrument, also developed by the WHOQOL group).

The results of the current study have the potential to establish the use of the WHOQOL-OLD module and the short forms as statistically reliable measures in a South African context with a view to aid the well-being of older people. Baseline data on older people's rating of their QoL in these settings will provide care providers as well as policy makers with a more holistic idea of what older people need in order to have QoL while living in institutions.

## Methodology

### Research method and design

A non-experimental, descriptive approach was followed, therefore no hypothesis is posed. A cross-sectional survey design was implemented for the study.

### The research instrument

#### Scale description

Under the auspices of the World Health Organization Quality of Life group, a collaborative effort amongst numerous researchers from various countries has led to the development of a measure focusing on the QoL in older population cohorts (29). The initial development of the generic WHOQOL measures of QoL occurred in 15 different centres worldwide. Two main generic instruments resulted; the WHOQOL-100, which consists of 24 facets grouped into 6 domains, and the WHOQOL-BREF which is a reduced 26-item version with four domains. The development of these instruments included a multidimensional and multicultural approach that suggested the assessment of physical, psychological, social relations, environmental, and overall QoL and health satisfaction domains (31). Controversy over the issue of whether the WHOQOL instruments that had been validated in younger adult populations were suitable for elderly samples led to the development of an add-on module for older people.

In the development of an add-on module, 22 centres around the world were involved in conducting focus groups with older people, their carers and other professionals working with older people (29) (South Africa was not one of the centres). These focus groups aimed to identify gaps in the original generic instruments which were relevant to QoL for older people. The outcome was the WHOQOL-OLD which is a self-reporting, 24-item and 6-facet quantitative instrument in English, which measures specific aspects of QoL as pertaining to older people. These facets include: sensory abilities (SAB); autonomy (AUT); past, present, and future activities (PPF); social participation (SOP); death and dying (DAD); and intimacy (INT).

Similar to other WHOQOL instruments (WHOQOL-100, WHOQOL-Bref), each item is scored on a Likert-type scale ranging from 1 to 5 with higher scores representing greater QoL. Items 1, 2, 6, 7, 8, 9, and 10 are reverse-coded items. The time period assessed in the module comprises the 2-week period prior to testing and the instrument is based on self-report.

#### Internal consistency

Cronbach's *α* as a measure of internal consistency demonstrated satisfactory values with an acceptable range from *α*=0.72 to *α*=0.88 for each facet score, while the total score displayed a consistency coefficient of *α*=0.89 (27).

### Research context and participants

The research context can be sketched against the backdrop of long-term care facilities that offer independent living, assisted living, and frail care living arrangements (6). The target population comprised all older people residing in long-term care facilities in Potchefstroom. According to Stats SA's mid-year population estimates in 2012, the North-West province has high numbers of older people in terms of land per province, therefore long-term care facilities in Potchefstroom were used as data collection sites.

Convenience sampling was used to obtain participants residing in these long-term care facilities in Potchefstroom. Older people of both genders above the age of 60 were acquired as participants. The only exclusion criteria that pertained to the study constituted cases where the older people was longer able to communicate congruently or was frail to the point where they needed full-time care. The latter were not included in the study in order to protect them from giving consent without comprehending what was entailed.

Participants belonged to the same subculture and shared various characteristics, for example, inhabiting a shared living space. Afrikaans was the mother tongue of all the participants, although all participants could also speak and fully understand English. A total of 176 willing participants of both genders between the ages of 61 and 95 years and who were still able to communicate congruently and fully understood the purpose of the research completed the questionnaires. Although some researchers suggest that structural equation modelling (SEM) and confirmatory factor analysis (CFA) should not be used on samples smaller than 200, others suggest minimum sample sizes between 100 and 200 (32). In addition, when researchers use reliable and valid measuring instruments (such as the WHOQOL-OLD), such high-quality measures may compensate for a smaller sample size (33). Hence, the sample size of 176 was deemed as acceptable for the purposes of the current study. The original English version of each item was available in the self-reported survey.

Biographical information pertaining to age, gender, language, ethnicity, gender, marital status, health status, and cognitive abilities was obtained. The mean age amongst participants was 77 years (SD=8.1) and the male-to-female ratio yielded 50(M):126(F). The majority (except for two) of participants constituted white South Africans; this notion corresponds with the explanation of trends in long-term care occupation in South Africa in the introduction of this study. Participants’ marital status did not form part of the selection criteria, although more than half were widowed at the time of research.

The majority of participants resided independently on the premises, implying they were still fully capable of taking care of themselves. On a self-rating, Likert-type scale (1=poor, 5=excellent), participants rated their health as average to good. Similarly, on a self-rating scale for cognitive abilities, participants rated their abilities as average to good.

### Research procedure and ethics

A thorough literature study were conducted which focused on the nature and impact of QoL of older people residing in long-term care facilities and screening instruments that have been used in the field. Permission to use the WHOQOL-OLD module within a South African context was obtained from the authors in the United Kingdom.

Ethical approval was obtained from the HREC with the following ethics number and title: NWU-00053-10-S1 ‘An exploration of enabling contexts’. Managers of facilities were contacted to negotiate them acting as mediators in the study, where after the aims and the process of the research were explained to them. The researcher and manager of each facility worked in close collaboration in order to make the distribution of questionnaires as well as the administration and collection thereof as hassle free as possible to the residents. All residents received a document which contained relevant information on the purpose and procedures of the study. They were assured of privacy and confidentiality. They were furthermore informed that they could withdraw from the study at any point and refuse to fill in surveys without any negative consequences. Questionnaires were numbered/coded, therefore the identity of participants was not revealed.

Older people living in the participating facilities were invited to the research by means of an accompanying letter 2 weeks prior to the actual date of commencement of data collection. An informed consent form attached to the questionnaire was posted to the residents in their on-site mailboxes. Willing participants had 2 weeks to complete the questionnaire and to place it in the red box provided by the researcher at the reception area of the facility. The boxes were clearly marked with the phrase ‘completed research forms’. The researcher collected the box on the date specified in the accompanying letter.

The population group of interest can be regarded as a vulnerable community. A possible decrease in reading abilities (sight) amongst older persons was considered and thus the questionnaire was made available/distributed in large print. No persons were excluded on the basis of race, disability, gender, language, religion, or sexual orientation. Possible risks of the study included possible fatigue and emotional turmoil to older people who had emotional reactions to some of the items in the questionnaire. However, no such incidents were reported.

### Statistical analysis

The questionnaires were coded into an Excel spreadsheet where after statistical analysis was performed. The current study analysed the correlation matrix during exploratory factor analysis (EFA). Principal axis factor analysis was employed to investigate the underlying structure of the latent variable. In order to determine the number of factors to be extracted, both parallel analysis (PA) (34) and the minimum average partial test (MAP) were used. These two techniques are viewed as the most reliable estimation methods with a statistical basis (34, 35). To determine whether the correlation matrix was factor analysable (36), Bartlett's Test of Sphericity had to be significant, while the Kaiser–Meier–Olkin measure of sampling adequacy had to be above the recommended value of 0.6 (37). Factor loadings of 0.3 were deemed as significant (38).

In order to evaluate the competing factor structures associated with the WHOQOL-OLD as well as the three short forms, the current study employed CFA. In so doing, the current study follows the suggestion by Van Prooijen and Van Der Kloot (39). In essence, they seem to suggest that ‘if CFA cannot confirm results of EFA on the same data, one cannot expect that CFA will confirm results of EFA in a different sample or population (39, p. 780)’. In addition to evaluating the factor structure suggested by EFA, comparable fit statistics for the original structure of the WHOQOL-OLD were obtained by means of CFA.

In estimating the reliability associated with the dimensions of the WHOQOL-OLD and the short versions, Cronbach's alpha (*α*) was employed. Reliability estimates that are 0.7 and higher are indicative of good reliability. However, estimates as low as 0.6 may be acceptable when conducting exploratory research (38).

The data were treated as continuous. Through analysing the covariance matrix, the data were assessed for normality (40). A test of multivariate normality was used to determine the skewness of data to be used during CFA (41). On the basis of the test of multivariate, the data were deemed skewed. The latter required the use of the robust maximum likelihood method of estimation.

All the analyses related to the CFA were conducted using LISREL 8.80 (41). Several fit indices were used, including the Satorra–Bentler Scaled Chi-square, Root Mean Square Error of Approximation (RMSEA), Comparative Fit Index (CFI), and the Goodness of Fit Index (GFI). Values close to 0.95 for GFI and CFI are considered indicative of a good model fit. It is suggested that values close to 0.06 are indicative of acceptable fit for RMSEA (42). In addition, Akaike's Information Criterion (AIC) can be used when comparing competing models, with smaller values indicating the better fitting model (43).

## Results

Table 1 provides the descriptive statistics associated with the current sample. The means and standard deviations of the original six-factor structure and the three short versions of the WHOQOL-OLD are also reported.

**Table 1:** Descriptive statistics

Variables	Frequency	
Male	50	
Female	126	
Variables	Mean	Standard deviation
Age	77	8.1
SAB	16.22	3.01
AUT	15.10	2.72
PPF	15.78	2.29
SOP	15.83	2.52
DAD	16.28	3.45
INT	15.63	3.46
WHOQOL-OLD Total Score (24 items)	94.83	11.64
WHOQOL-OLD Short Version 1 (six items)	23.24	3.33
WHOQOL-OLD Short Version 2 (six items)	23.47	3.44
WHOQOL-OLD Short Version 3 (six items)	23.63	3.05

SAB, sensory abilities; AUT, autonomy; PPF, past, present, and future activities; SOP, social participation; DAD, death and dying; INT, intimacy.

From Table 2, it is evident that there are three eigenvalues from the dataset that are bigger than the eigenvalues (95th percentile) from the random dataset.

**Table 2:** Parallel analysis results

Variables	Real-data eigenvalues	Mean of random eigenvalues	95 percentile of random eigenvalues
1	32.0	8.9	9.8
2	11.5	8.2	8.8
3	10.7	7.6	8.1
4	6.7	7.1	7.6
5	5.9	6.7	7.1
6	4.0	6.3	6.7
7	3.8	5.9	6.3
8	3.4	5.6	5.9
9	3.1	5.2	5.6
10	2.7	4.9	5.2
11	2.4	4.6	4.8
12	2.1	4.2	4.5
13	2.0	3.9	4.2
14	1.9	3.6	3.9
15	1.5	3.3	3.6
16	1.4	2.9	3.3
17	1.3	2.6	3.0
18	1.1	2.3	2.6
19	0.8	1.9	2.3
20	0.7	1.6	2.0
21	0.5	1.2	1.6
22	0.2	0.9	1.3
23	0.2	0.5	0.9
24	0.0	0.0	0.0

The results of the MAP (Table 3) show the smallest average partial to be 0.02311. This value is associated with the second dimension. Hence, based on the results of both PA and MAP, both three- and two-dimensional structures can be investigated based on EFA results.

**Table 3:** Minimum average partial test (MAP results)

Dimensions	Average partial
1	0.03242
2	0.02311
3	0.02356
4	0.04074
5	0.09252
6	0.37147
7	0.99999

Table 4 reports on the significant factor loadings associated with the two-dimensional factor structure – as suggested by the MAP test. Factor 1 contains items representing Intimacy. The items loading on Factor 2 seem to represent developmental tasks required by individuals to experience a high QoL during their old age. Both these two factors have reliabilities exceeding 0.80. This two-dimensional solution had a Kaiser–Meier–Olkin measure of sampling adequacy (KMO) of 0.88. In addition, Bartletts’ Test of Sphericity was significant.

**Table 4:** Factor loadings for the two-dimensional structure of the WHOQOL-OLD

Variables	F 1	F 2
1		0.625
2		0.745
3		0.644
4		0.600
5		0.392
6		0.439
7		0.411
8		0.490
9		0.384
10		0.606
11		0.589
12		0.667
13		0.569
14		0.512
15		0.544
16		0.607
17		0.546
18		0.615
19		0.608
20		0.655
21	0.716	
22	0.739	
23	0.907	
24	0.903	
Reliability	0.92	0.92

The items that had significant factor loadings, associated with the three-dimensional structure, are reported in Table 5. The three-dimensional solution had a KMO of 0.84. In addition, Bartletts’ Test of Sphericity was significant. Factor 1 contains items representing Death and Dying. The items loading on Factor 2 represent Intimacy. Factor 3 seems to represent the developmental tasks required by individuals to experience a high QoL during their old age. All three the identified factors have reliabilities exceeding 0.80.

**Table 5:** Factor loadings for the three-dimensional structure of the WHOQOL-OLD

Items	F 1	F 2	F 3
1			0.537
2			0.690
3			0.579
4			0.528
5			0.354
6	0.790		
7	0.817		
8	0.735		
9	0.596		
10			0.505
11			0.615
12			0.647
13			0.446
14			0.480
15			0.434
16			0.561
17			0.468
18			0.552
19			0.587
20			0.626
21		0.713	
22		0.721	
23		0.902	
24		0.893	
Reliability	0.85	0.92	0.89

To allow for meaningful comparisons, the current study used CFA to evaluate three different conceptualisations of the WHOQOL-OLD. The original six-dimensional structure was compared with the factor structures proposed by PA (three factors) as well as the MAP (two factors). It is clear that the original factor structure exhibits better fit than the other two models – see Table 6. The values associated with the RMSEA, CFI, and SRMR all indicate acceptable model fit. However, the two-factor conceptualisation of the WHOQOL-OLD exhibits poor levels of fit as evident from the values associated with the RMSEA, SRMR, and CFI. The indices associated with the three-dimensional structure points to a model with reasonable fit.

**Table 6:** Summary of goodness-of-fit statistics (WHOQOL-OLD module)

	Original factor structure	Two-dimensional structure	Three-dimensional structure
S-B χ^2^	339.05	713.00	576.39
Df	237	251	249
RMSEA	0.050 (0.037; 0.061)	0.111 (0.094; 0.111)	0.075 (0.065; 0.085)
SRMR	0.070	0.104	0.081
CFI	0.97	0.88	0.94
AIC	465.05	1015.36	597.80

S-B χ^2^, Satorra–Bentler Scaled Chi-square; df, degrees of freedom; RMSEA, root mean square error of approximation; SRMR, standardised root mean square residual; CFI, comparative fit index; AIC, Akaike's information criterion.

The goodness-of-fit statistics associated with the short versions of the WHOQOL-OLD are reported in Table 7. It is clear that all three measurement models exhibit good levels of fit to the data – especially in terms of the values associated with the RMSEA, CFI, and SRMR. Version 3 seems to be the best fitting model due to it having the lowest value associated with AIC.

**Table 7:** Summary of goodness-of-fit statistics (short versions of the WHOQOL-OLD module)

	Version 1	Version 2	Version 3
S-B χ^2^	13.899	10.910	10.806
df	9	9	9
RMSEA	0.056 (0.000; 0.110)	0.083 (0.000; 0.096)	0.034 (0.000; 0.095)
SRMR	0.046	0.047	0.041
CFI	0.98	0.98	0.98
AIC	37.89	34.91	34.81

S-B χ^2^, Satorra–Bentler Scaled Chi-square; df, degrees of freedom; RMSEA, root mean square error of approximation; SRMR, standardised root mean square residual; CFI, comparative fit index; AIC, Akaike's information criterion.

The standardised factor loadings and errors are reported in Table 8 for the measurement model depicting the original factor structure of the WHOQOL-OLD. It is evident that both the AUT and PPF dimensions have the lowest reliabilities in comparison with the other dimensions. However, they are still above the acceptable level of 0.6.

**Table 8:** Standardised factor loadings and errors: original factor structure

Items	SAB	AUT	PPF	SOP	DAD	INT	Error
20	0.61						0.63
1	0.60						0.65
2	0.82						0.32
10	0.64						0.59
3		0.74					0.45
4		0.73					0.47
5		0.55					0.70
11		0.58					0.66
12			0.70				0.50
13			0.53				0.72
15			0.53				0.72
19			0.70				0.51
14				0.58			0.66
16				0.75			0.43
17				0.64			0.59
18				0.66			0.57
6					0.76		0.42
7					0.82		0.33
8					0.79		0.38
9					0.59		0.66
21						0.75	0.44
22						0.78	0.39
23						0.91	0.17
24						0.91	0.18
*α*	0.759	0.726	0.716	0.740	0.817	0.902	

SAB, sensory abilities; AUT, autonomy; PPF, past, present, and future activities; SOP, social participation; DAD, death and dying; INT, intimacy.

The phi matrix, reporting the correlations amongst the six dimensions of the WHOQOL-OLD, is presented in Table 9. It is evident that DAD and INT have low correlations with some of the other five dimensions.

**Table 9:** Phi matrix: original factor structure

	1	2	3	4	5	6
1. SAB	1.00					
2. AUT	0.51	1.00				
3. PPF	0.62	0.81	1.00			
4. SOP	0.55	0.60	0.99	1.00		
5. DAD	0.21	0.26	0.33	0.27	1.00	
6. INT	0.13	0.31	0.55	0.48	0.16	1.00

SAB, sensory abilities; AUT, autonomy; PPF, past, present, and future activities; SOP, social participation; DAD, death and dying; INT, intimacy.

## Discussion

The aim of the current study was a partial validation (i.e. the assessment of the factor structure as and the internal consistency reliability) of the WHOQOL-OLD and its three shorter versions. Encouraging results were found related to original factor structure of the WHOQOL-OLD. The data obtained from the current sample seem to fit the model of the instrument well, especially when considering the goodness-of-fit statistics (RMSEA=0.050; CFI=0.97; SRMR=0.070). All three these indices are indicative of a well-fitting model. The reliabilities associated with the six dimensions also point to a reliable instrument. However, the reliabilities associated with the AUT (*α*=0.726) and PPF (*α*=0.716) may leave room for some improvement.

Using a large sample (*n*=5566) from 20 different countries, the developers of the WHOQOL-OLD found the following goodness-of-fit statistics (RMSEA=0.052; CFI=0.939) associated with a measuring instrument consisting of six dimensions (30). It is clear that the current study echoes those results obtained by Power and Schmidt, albeit with slightly better fit.

The estimates of reliability found in the current study are slightly lower than that obtained by Power and Schmidt (30): Sensory Abilities (*α*=0.76 vs. *α*=0.84), Social Participation (*α*=0.74 vs. *α*=0.79), Death and Dying (*α*=0.82 vs. *α*=0.84), and Past, Present, and Future Activities (*α*=0.72 vs. *α*=0.74). The current study found that the following dimensions had slighter higher reliabilities: Intimacy (*α*=0.90 vs. *α*=0.88), Autonomy (*α*=0.73 vs. *α*=0.72).

In a sample of 400 participants, Bowling (44) reported a very low reliability (*α*=0.40) associated with all 24 items of the WHOQOL-OLD. However, the same researcher found a much more acceptable reliability (0.845) in another sample (*n*=536). The current study obtained a higher reliability (*α*=0.882) with a smaller sample (*n*=176).

Analysing the phi matrix (the intercorrelations between the sub-dimensions), it is evident that the majority are moderately related. However, the Death and Dying and Intimacy dimensions show much lower correlations with the other sub-dimensions. A similar result was obtained by the developers of the WHOQOL-OLD with regard to the Death and Dying dimension (29, 30). Interestingly, Chachamovich et al. (28) found that the Death and Dying dimension had inadequate fit to their model.

In short, the current study seems to support the findings of the developers of the WHOQOL-OLD in terms of the original factor structure of the WHOQOL-OLD (30).

Researchers also explored three shorter versions of the WHOQOL-OLD instrument (27). These authors found acceptable reliabilities associated with Version 1 (*α*=0.68), Version 2 (*α*=0.68), and Version 3 (*α*=0.65). Findings reported in the current study show a slightly lower reliability for Version 1 (*α*=0.65). In contrast to the findings of Fang et al. (27), Version 2 (*α*=0.61), and Version 3 (*α*=0.57) had lower reliability estimates. This is especially true for Version 3, which leaves room for improvement which can be done through more studies on the validation of these three versions of the short-form WHOQOL-OLD module.

The developers of the three short versions did not obtain goodness-of-fit statistics associated with each of these three measurement models (27). The current study contributes to the current literature by providing these goodness-of-fit statistics. It is clear that although the three versions had lower estimates of reliability, the data exhibited excellent fit to the theory – especially when looking at the values associated with the RMSEA, CFI, and SRMR. In addition, AIC seems to suggest that Version 3 of the short WHOQOL-OLD provides a better fitting model than the other two versions.

In addition, PA and the MAP suggested two competing conceptualisations of the WHOQOL-OLD. The current sample seems to suggest that the three-dimensional model is a better conceptualisation when compared with the two-dimensional model – based on the value of AIC. Only the three-dimensional model exhibited acceptable levels of fit, although it is not as good as that of the original structure proposed by Power and Schmidt (30).

### Recommendations and limitations

South Africa has 11 official languages. A study by Wissing et al. (45) describes language as the medium through which cultural meaning is created. It is recommended that future studies could consider validation of the WHOQOL-OLD modules in the mother tongue of the people involved.

Depending on the needs of the researchers, they may use either the original WHOQOL-OLD questionnaire with its 24 items, or Version 3 of the three short versions of this instrument. It is suggested that when researchers want to cover the breadth of QoL in older adults that the WHOQOL-OLD be used. This will enable them to investigate the impact of six dimensions on various important outcomes associated with QoL.

However, when researchers are concerned about the impact that the length of the WHOQOL-OLD may have on the responses of older adults, then the short version could be used. It is furthermore recommended that the two competing conceptualisations of the original WHOQOL-OLD be further investigated by other researchers to determine the applicability of these factor structures.

A limitation of the study is that findings cannot be generalised to other ethnic groups or older people in other contexts due to sample size and cultural diversities in South Africa. However, the sample was deemed sufficient for the partial validation (i.e. the assessment of the factor structure as and the internal consistency reliability) of the WHOQOL-OLD and its shorter versions, as applied to Afrikaans-speaking older people who were equally proficient in English and shared an individualistic way of living with Western contexts, which were the primary objectives of this study.

## Conclusions

The overarching aim of determining the psychometric properties of the WHOQOL-OLD was to establish whether the instrument could reliably be used within the South African context. A reliable measure with good psychometric properties will yield new knowledge in the area of QoL and might direct future research efforts and put current resources in residential care facilities to better use. It is hoped that a better understanding of the QoL of older people as the result of utilising this instrument on a larger scale will inform policy makers, the management structures of facilities, care givers, family members of the residents, and the older people residing in these facilities on how to make decisions to promote their overall QoL.
